# Anatomy and neuro-pathophysiology of the cough reflex arc

**DOI:** 10.1186/2049-6958-7-5

**Published:** 2012-06-18

**Authors:** Mario Polverino, Francesca Polverino, Marco Fasolino, Filippo Andò, Antonio Alfieri, Francesco De Blasio

**Affiliations:** 1 Lung Diseases Institute, Medical Sciences Department, ASL Salerno, Viale S. Francesco, Nocera Inferiore (Salerno), Italy; 2 Brigham and Women’s Hospital and Harvard Medical School, 45 Francis St, Boston, MA, USA; 3 Neurology Institute, Medical Sciences Department, ASL Salerno, Viale S. Francesco, Nocera Inferiore (Salerno), Italy; 4 Lung Diseases Institute, University of Messina Medical School, Messina, Italy; 5 Respiratory Medicine and Pulmonary Rehabilitation Section, Clinic Center, Via Cintia, 80126, Naples, Italy

**Keywords:** Cough, C-Fibers, Rapidly adapting receptors, Reflex arc, Slowly adapting stretch receptors

## Abstract

Coughing is an important defensive reflex that occurs through the stimulation of a complex reflex arc. It accounts for a significant number of consultations both at the level of general practitioner and of respiratory specialists. In this review we first analyze the cough reflex under normal conditions; then we analyze the anatomy and the neuro-pathophysiology of the cough reflex arc. The aim of this review is to provide the anatomic and pathophysiologic elements of evaluation of the complex and multiple etiologies of cough.

## Review

Cough is one of the most common symptoms for which outpatient care is asked [1], accounting for up to 40% of the practice activity [2]. Coughing is an important defensive reflex that enhances clearance of secretions and particulates from the airways and protects from aspiration of foreign materials occurring as a consequence of aspiration or inhalation of particulate matter, pathogens, accumulated secretions, postnasal drip, inflammation, and mediators associated with inflammation. Under normal conditions cough serves an important protective role in the airways and lungs, but in some conditions it may become excessive and nonproductive, and is troublesome and potentially harmful to the airway mucosa. These contrasting consequences of coughing can be attributed to the parallel afferent pathways regulating this important defensive reflex of the airways.

Each cough occurs through the stimulation of a complex reflex arc. This is initiated by the irritation of cough receptors which are found in the trachea, main carina, branching points of large airways, and more distal smaller airways; also, they are present in the pharynx. Laryngeal and tracheobronchial receptors respond to both mechanical and chemical stimuli. Chemical receptors sensitive to acid, heat, and capsaicin-like compounds trigger the cough reflex via activation of the type 1 vanilloid (capsaicin) receptor [3-5]. In addition, more airway receptors are in the external auditory canals, eardrums, paranasal sinuses, pharynx, diaphragm, pleura, pericardium, and stomach. These are probably mechanical receptors only, which can be stimulated by triggers such as touch or displacement.

Impulses from stimulated cough receptors traverse an afferent pathway via the vagus nerve to a ‘cough center’ in the medulla, which itself may be under some control by higher cortical centers. The cough center generates an efferent signal that travels down the vagus, phrenic, and spinal motor nerves to expiratory musculature to produce the cough.

Therefore, the **cough reflex arc** is constituted by:

1. **Afferent pathway:** Sensory nerve fibers (branches of the vagus nerve) located in the ciliated epithelium of the upper airways (pulmonary, auricular, pharyngeal, superior laryngeal, gastric) and cardiac and esophageal branches from the diaphragm. The afferent impulses go to the medulla diffusely.

2. **Central Pathway (cough center):** a central coordinating region for coughing is located in the upper brain stem and pons.

3. **Efferent pathway:** Impulses from the cough center travel via the vagus, phrenic, and spinal motor nerves to diaphragm, abdominal wall and muscles. The nucleus retroambigualis, by phrenic and other spinal motor nerves, sends impulses to the inspiratory and expiratory muscles; and the nucleus ambiguus, by the laryngeal branches of the vagus to the larynx.

The terminations of the vagal afferents are found in abundance in the airway mucosa and in the airway wall from the upper airways to the terminal bronchioles and lung parenchyma. Afferent neuronal subtypes can be identified based on their physicochemical sensitivity, adaptation to sustained lung inflation, neurochemistry, origin, myelination, conduction velocity (A-fiber, > 3 m/s; C-fiber, < 2 m/s), and sites of termination in the airways. These attributes can be used to identify at least three broad classes of airway afferent nerves:

1. Rapidly Adapting Receptors (RAR)

2. Slowly Adapting Stretch Receptors (SARs)

3. C-Fibers

### Rapidly adapting receptors (RAR)

Functional studies of RARs suggest that they terminate within or beneath the epithelium of both intrapulmonary and extrapulmonary airways, but primarily the intrapulmonary airways. RARs are differentiated from other airway afferents by their rapid adaptation (in 1 - 2 seconds) to sustained lung inflations [6-19]. Other distinguishing properties of RARs include their sensitivity to lung collapse and/or lung deflation, their responsiveness to alterations in dynamic lung compliance (and thus their sensitivity to bronchospasm), and their conduction velocity (4 to 18 m/s), which is suggestive of myelinated axons. The sustained activation of RARs produced by dynamic lung inflation, bronchospasm, or lung collapse indicates that the adaptation of RARs is not attributable to an electrophysiologic adaptation. Perhaps RARs are thus better defined as dynamic receptors that respond to changes in airway mechanical properties (e.g. diameter, length, and interstitial pressures). RARs are sporadically active throughout the respiratory cycle, are activated by the dynamic mechanical forces accompanying lung inflation and deflation, and become more active as the rate and volume of lung inflation increase. RARs are activated by stimuli that evoke bronchospasm or obstruction resulting from mucus secretion or edema. Substances such as histamine, capsaicin, substance P, and bradykinin activate RARs in a way that can be markedly inhibited or abolished by preventing the local end-organ effects that these stimuli produce (e.g. bronchospasm and mucus secretion). RAR activation initiates reflex bronchospasm and mucus secretion through parasympathetic pathways. RARs can also respond to stimuli that evoke cough and fulfill many criteria for mediating cough. Further evidence for their role in coughing comes from studies of vagal cooling, which blocks cough at temperatures that selectively abolish activity in myelinated fibers (including RARs) while preserving C-fiber activity. RARs may act synergistically with other afferent nerve subtypes to induce coughing.

### Slowly adapting stretch receptors (SARs)

SARs are highly sensitive to the mechanical forces that are put on the lung during breathing. SAR activity increases during inspiration and peaks just prior to the initiation of expiration [13]. SARs are thus thought to be the afferent fibers involved in the Hering-Breuer reflex, which terminates inspiration and initiates expiration when the lungs are adequately inflated. SARs can be differentiated from RARs in some species based on action potential conduction velocity, and in most species by their lack of adaptation to sustained lung inflations. SARs may also be differentially distributed throughout the airways: they appear to terminate primarily in the intrapulmonary airways. SARs also differ from RARs with respect to the reflexes they precipitate. SAR activation results in the central inhibition of respiration and the inhibition of the cholinergic drive to the airways, leading to decreased phrenic nerve activity and decreased airway smooth muscle tone (due to a withdrawal of cholinergic nerve activity) [14]. The sensory terminals of SARs assume a complex and varying position within the airway wall: most of these SARs are found in the peripheral airways (associated with alveoli or bronchioles). Occasionally, SAR dendritic arbors are associated with the bronchiolar smooth muscle. SARs may facilitate coughing by a central cough network via activation of brainstem second-order neurons of the SAR reflex pathway.

### C-fibers

The majority of afferent nerves innervating the airways and lungs are unmyelinated C-fibers. They are similar in many ways to the unmyelinated somatic sensory nerves innervating the skin, skeletal muscle, joints, and bones that respond to noxious chemical and mechanical stimuli (called nociceptors). In addition to their conduction velocity (< 2 m/s), airway vagal afferent C-fibers are distinguished from RARs and SARs by their relative insensitivity to mechanical stimulation and lung inflation. C-fibers are further distinguished from RARs by the observation that they are directly activated by bradykinin and capsaicin, not indirectly through effects on smooth muscle or the airway vasculature. Moreover, prostaglandin E2, adrenaline, and adenosine, which by bronchodilating the airways might inhibit RAR activation by bradykinin and capsaicin, actually sensitize C-fibers to capsaicin and bradykinin through direct effects on their peripheral nerve terminals [15-17]. Morphologic studies in rats and in guinea pigs have revealed that afferent C-fibers innervate the airway epithelium as well as other effector structures within the airway wall. C-fibers may synthesize neuropeptides that are subsequently transported to their central and peripheral nerve terminals. C-fibers are generally quiescent throughout the respiratory cycle but are activated by chemical stimuli such as capsaicin, bradykinin, citric acid, hypertonic saline solution, and sulfur dioxide (SO_2_). Reflex responses evoked by C-fiber activation include increased airway parasympathetic nerve activity, and the chemoreflex, characterized by apnea (followed by rapid shallow breathing), bradycardia, and hypotension. Stimulants of C-fibers such as capsaicin, bradykinin, SO_2_, and citric acid evoke cough in conscious animals and in humans, and capsaicin desensitization abolishes citric acid-induced coughing in guinea pigs.

Sex-related differences in cough reflex sensitivity explain the observation that women are more likely than men to develop chronic cough [18-20].

The mechanical events of a cough can be divided into three phases [21]:

1. Inspiratory phase: Inhalation, which generates the volume necessary for an effective cough.

2. Compression phase: Closure of the larynx combined with contraction of muscles of chest wall, diaphragm, and abdominal wall result in a rapid rise in intrathoracic pressure.

3. Expiratory phase: The glottis opens, resulting in high expiratory airflow and the coughing sound. Large airway compression occurs. The high flows dislodge mucus from the airways and allow removal from the tracheobronchial tree.

The specific pattern of the cough depends on the site and type of stimulation. Mechanical laryngeal stimulation results in immediate expiratory stimulation (sometimes termed the expiratory reflex), probably to protect the airway from aspiration; stimulation distal to the larynx causes a more prominent inspiratory phase, presumably to generate the airflow necessary to remove the stimulus.

During vigorous coughing, intrathoracic pressures may reach 300 mm Hg and expiratory velocities approach 800 kilometers per hour [22]. While these pressures and velocities are responsible for the beneficial effects of cough on mucus clearance, they are also responsible for many of the complications of cough, including exhaustion, self-consciousness, insomnia, headache, dizziness, musculoskeletal pain, hoarseness, excessive perspiration, urinary incontinence [23]. Cough-induced rib fractures are another painful and potentially serious complication of chronic cough. Fractures often involve multiple ribs, particularly ribs five through seven. Women with decreased bone density are at the greatest risk of this complication; however, fractures can occur in patients with normal bone density as well [24].

A nonproductive cough is a well-recognized complication of treatment with angiotensin converting enzyme (ACE) inhibitors, occurring in up to 15% of patients treated with these agents [25]. Although the pathogenesis of the cough is not known with certainty, it has commonly been hypothesized that accumulation of bradykinin, which is normally degraded in part by ACE, may stimulate afferent C-fibers in the airway [26].

The important observation that cough does not appear to occur with increased frequency in patients treated with angiotensin II receptor antagonists (which do not increase kinin levels) is consistent with the kinin hypothesis.

Lesions that compress the upper airway, including arteriovenous malformations and retrotracheal masses, may present with chronic cough [27-29]. Cough can also be a symptom of tracheobronchomalacia, which results from loss of rigid support of the large airways and inspiratory collapse, and is usually seen in conjunction with obstructive lung disease in patients with a history of cigarette smoking [30].

Laryngeal sensory neuropathy has been identified as the cause of chronic cough in 18 of 26 patients with acute onset of cough that was often associated with laryngospasm or throat clearing [31]. Chronic tonsillar enlargement has been proposed as a cause of chronic cough, but clinical evidence of this association is limited. One series of 236 patients referred for evaluation in a specialized clinic noted tonsillar enlargement in the absence of other known causes of chronic cough in 8 (3.4%) individuals [32]. Following tonsillectomy, these patients had decreased cough sensitivity and significantly improved symptom control. At this moment, these observations need further investigations.

Irritation of the external auditory canal by impacted foreign bodies or cerumen is another unusual cause of chronic dry cough [33]. The etiology of the ‘ear-cough’ (or oto-respiratory) reflex is related to stimulation of the auricular branch of the vagus nerve (Arnold's nerve) [34,35]. Another rare cause of chronic cough is Holmes-Adie syndrome due to autonomic dysfunction affecting the vagus nerve [36] patients present anisocoria, abnormal deep tendon reflexes, and patchy areas of hyperhidrosis or anhidrosis.

In adults, habit (also known as ‘psychogenic’) cough may rarely be the cause of a chronic cough that remains troublesome despite a thorough evaluation, including ruling out tic disorders.

Differences among several sites from which cough stimuli can originate may result in variations in the sounds and patterns of coughing.

Laryngeal stimulation produces a choking type of cough without a preceding inspiration.

Inadequate mucociliary clearance mechanisms (as in bronchiectasis or cystic fibrosis) may produce a pattern of coughing with less violent acceleration of air and a sequence of interrupted expirations without any intervening inspiration.

Awareness of cough varies considerably: it can be distressing when it appears suddenly, especially if associated with discomfort due to chest pain, dyspnea, or copious secretions, while a cough that develops over decades (e.g. in a smoker with chronic bronchitis) may be hardly noticeable or may be considered normal by the patient.

Since **c**ough is an important defensive reflex, required to maintain the health of the lungs, people who do not cough effectively are at risk of atelectasis, recurrent pneumonia, and chronic airways disease from aspiration and retention of secretions. Many disorders can impair the ability to cough effectively, which may result in persistent cough. The elderly, newborns, lung transplant recipients, and patients with paralysis or neuromuscular disorders have a poorly developed and/or compromised cough reflex, and are rendered highly susceptible to lung infections and aspiration pneumonia. Patients with paralysis or neuromuscular disorders (including rib fractures) and chest wall deformities may not generate the flows necessary for effective clearance of secretions due to defective "pump" mechanisms [37]. Also patients with reduced function of the abdominal wall musculature are particularly at risk for ineffective cough. Patients with tracheo-bronchomalacia ("floppy" airways), or with obstructive airways diseases, often do not generate the high flow rates needed for effective clearance of secretions. Individuals with laryngeal disorders, including those with tracheostomies, may not achieve sufficient laryngeal closure to generate the increased intrathoracic pressures necessary for an effective cough [38,39].

## Conclusions

Based on this complex mechanism, the treatment often requires an early symptomatic approach in order to prevent the vicious cycle of cough perpetuating cough [40] and a chronic disorder which has been reported to be the fifth most common complaint seen by primary care physicians in the world and the third in Italy [41].

## Competing interests

The authors declare that they have no competing interests.
